# Cellular remodeling of lipedema affected subcutaneous adipose tissue

**DOI:** 10.3389/fcell.2026.1893552

**Published:** 2026-10-01

**Authors:** Sabrina Gohlke, Francisco Garcia-Carrizo, Robin Upmeyer, Tim J. Schulz

**Affiliations:** 1 Department of Adipocyte Development and Nutrition, German Institute of Human Nutrition Potsdam-Rehbrücke, Nuthetal, Germany; 2 German Center for Diabetes Research (DZD), Neuherberg, Germany; 3 Institute of Nutritional Science, Potsdam-Rehbrücke, University of Potsdam, Nuthetal, Germany

**Keywords:** adipocytes, adipogenic stem and progenitor cells, inflammation, lipedema, surface marker proteins

## Abstract

A progressive disease, lipedema is characterized by bilaterally symmetrical and disproportional accumulation of subcutaneous adipose tissue (SAT) in the extremities, leading to severe pain, inflammation, and mobility issues. Lipedema predominantly affects women in all stages of life and treatment options are currently limited as patients’ conditions can only be improved to a limited extent by diet or exercise. The pathogenesis of lipedema remains poorly understood and a reliable diagnostic marker is currently missing. Disease progression is marked by stage-dependent morphological adipose tissue changes, such as adipocyte hypertrophy and stage-dependent fibrosis, accompanied by an elevated proportion of anti-inflammatory macrophages. The molecular mechanisms regulating these compositional and structural changes in adipose tissue, and to what extent this altered response is cause or consequence of the disease, remain unclear. Therefore, the analysis of disease-related changes, with a focus on the cellular dynamics and tissue composition changes may help to distinguish lipedema more reliably from adiposity-related comorbidities.

## Introduction: distinction of lipedema from obesity

Lipedema is a painful disease that affects up to ∼12% of women in different stages of life and is presumably triggered by hormonal changes (Wold et al., 1951; Kruppa et al., 2020). Its main characteristics are disproportional, bilateral accumulation of SAT in the extremities, mobility issues, and resistance to multimodal therapy for weight loss (Herbst, 2012). Although some studies have reported a certain level of genetic inheritance (Child et al., 2010; Klimentidis et al., 2023; Morgan et al., 2024), the causes of the disease and its pathogenesis remain incompletely understood. Treatment options are very limited, and only liposuction provides patients with reliable relief (Ghods and Kruppa, 2018; Peprah and MacDougall, 2019). As lipedema symptoms are frequently misdiagnosed as obesity, patients commonly endured long periods of conservative treatments that have little effect on lipedema until liposuction is performed. Currently, no reliable biomarkers are available for an unambiguous diagnosis of the disease. Although lipedema, especially in higher disease stages, can be associated to increased body weight and obesity, clear distinctions exist between patients with lipedema and obesity (Figure 1). The most common criterion is the bilaterally symmetrical and disproportionate hypertrophy of adipose tissue on the limbs, while sparing hands and feet, which is accompanied by a high degree of inflammation. Moreover, patients report easy bruising, pain upon pressure and reduced mobility due to occurrence of edema. Somewhat paradoxically, although patients suffer from a significant reduction in quality of life, blood lipid profiles and insulin sensitivity appear to be improved compared to healthy individuals with matching body mass index (BMI); (Nankam et al., 2022).

**FIGURE 1:**
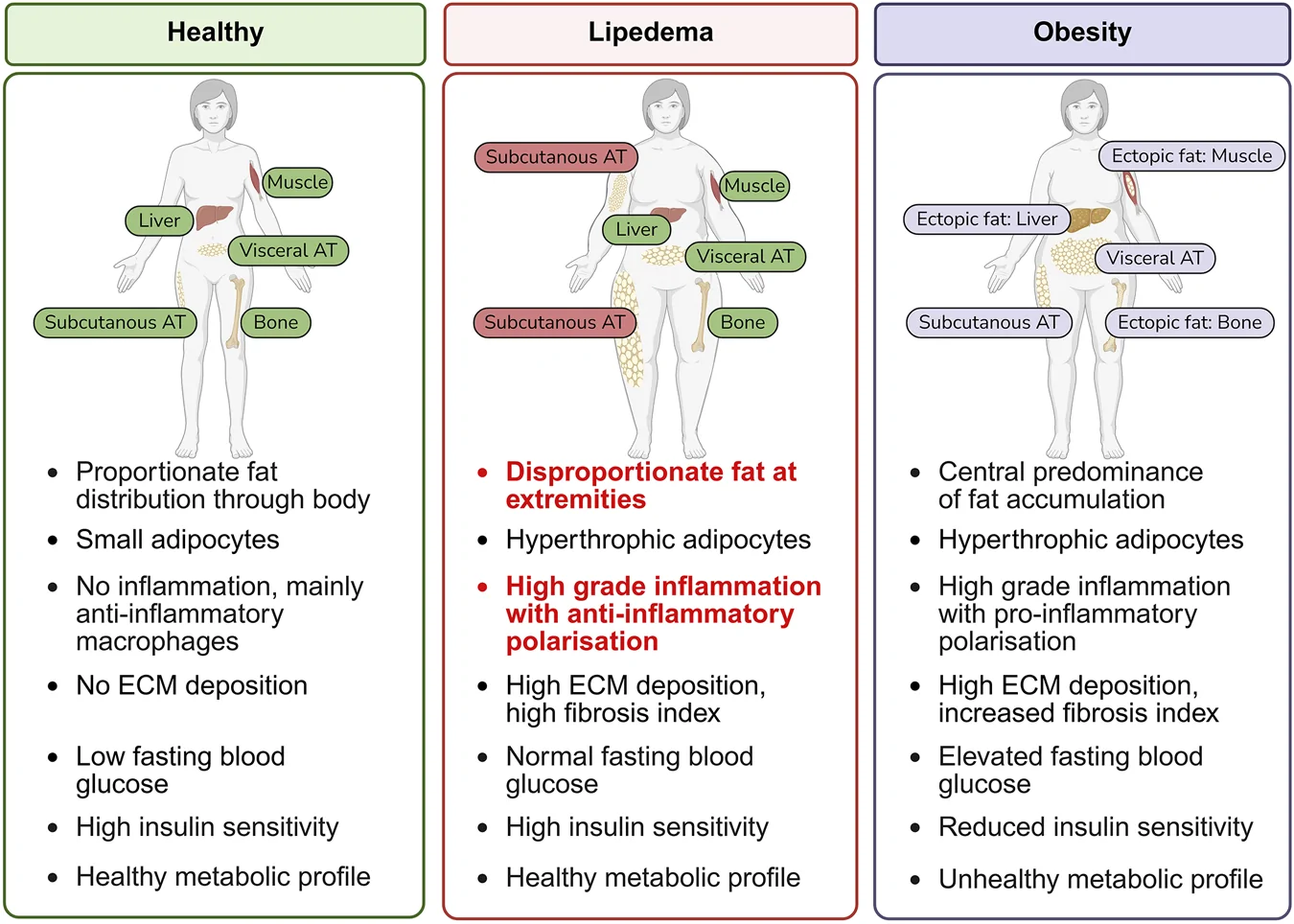
Patients with lipedema display a distinct pathology profile compared to patients with obesity. Compared to healthy, normal-weight individuals and patients suffering from obesity which is commonly linked to uniform expansion of all subcutaneous and visceral adipose tissue (AT) depots as well as ectopic lipid accumulation in various anatomical sites (purple), distinct anatomical sites are affected in patients with lipedema, who suffer from disproportional fat accumulation at the extremities and high-grade inflammation (red). However, while obese individuals are typically insulin resistant and show marked dyslipidemia, lipedema is accompanied by a healthier metabolic profile. (Wold et al., 1951; Herbst, 2012; Nankam et al., 2022; Kruppa et al., 2023). Created in BioRender https://BioRender.com/jufcl7k *.*

## Molecular signatures of subcutaneous adipose tissue in health and disease

White adipose tissue (WAT) depots are the primary sites of energy storage in the body. They are organized in discrete anatomical localizations that have been subdivided into subcutaneous and visceral depots, the latter of which typically surround the organs of the abdominal cavity. Additionally, WAT is an important endocrine organ with key functions in whole body metabolic homeostasis. Obesity occurs when calorie intake exceeds energy expenditure. While the expansion of visceral depots mainly occurs due to increases in adipocyte size, i.e., hypertrophy, subcutaneous depots are commonly linked to hyperplasia, i.e., the recruitment of additional adipocytes from proliferating tissue-resident progenitor cells. Once adipocyte and tissue volume are at capacity, ectopic fat storage occurs in other organs like the liver, muscle and pancreas, leading to organ steatosis (Olefsky and Glass, 2010).

Adipogenesis is driven by several transcription factors like peroxisome proliferator-activated receptor gamma (*PPARG*) and members of the CCAAT/enhancer-binding protein family (*C/EBP*), leading to the formation of mature adipocytes that secrete adipokines like adiponectin (ADIPOQ) and leptin (LEP); (Rosen et al., 1999). During obesity, hypertrophic adipocytes become hypoxic, producing pro-inflammatory cytokines like tumor necrosis factor alpha (TNFα) and interleukin 6 (IL6) and secreting increased amounts of LEP, while releasing decreasing amounts of ADIPOQ (Hotamisligil et al., 1993). Consequently, pro-inflammatory macrophages are recruited, accumulating around dying adipocytes in typical crown-like structures, and thereby further amplifying the release of cytokines that induce insulin resistance and promote lipolysis (Weisberg et al., 2003; Cinti et al., 2005; Olefsky and Glass, 2010).

To describe the molecular signature of lipedema is challenging, as the published literature is often contradictory. It is clear that excessive SAT accumulates in the extremities, but there is currently no consensus on whether this is caused by hyperplasia (Suga et al., 2009) or hypertrophy (Felmerer et al., 2020a; Kruppa et al., 2023). Further, disease-specific effects on the expression of adipocyte-specific markers also seem to vary when comparing the results of *in vitro* studies that examined gene expression and lipid accumulation following induction of adipogenic differentiation of patient-derived adipogenic progenitor cells. Several groups report that the expression of adipocyte gene markers such as *LEP, ADIPOQ* and *PPARG* is not affected (Felmerer et al., 2020a), or even decreased secretion of ADIPOQ and LEP in patients with lipedema (Bauer et al., 2019). Moreover, there are also studies describing increased *PPARG* or *LEP* gene expression levels and higher levels of circulating ADIPOQ in plasma of patients with lipedema in comparison to age- and BMI matched women (Al-Ghadban et al., 2020a; Al-Ghadban et al., 2020b; Nankam et al., 2022). These discrepancies could arise for different reasons: First, patient selection is crucial for the study outcome. Currently, diagnosis of patients depends on the subjective description of the patients’ symptoms and the assessment of the treating physician, which is based on partially subjective clinical parameters and after excluding alternative diagnoses (Kruppa et al., 2020). When comparing different studies, it is noticeable that age, BMI and stage distribution of the included patients are variable (Table 1). Reasons for this might stem from the varying onset of the disease following hormonal changes, e.g., after puberty, pregnancy, or menopause and the progressive nature, as lipedema-related changes of affected regions are highly stage-dependent (Figure 2). In stage I, the skin surface remains smooth, although palpable nodules are formed in SAT of the thigh region. The adipocyte size appears not significantly different from BMI matched individuals but the fibrosis index is higher in lipedema affected tissue and endothelia appear thickened at his stage (Kruppa et al., 2023; Al-Ghadban et al., 2026). In the second stage, the skin’s surface becomes increasingly uneven, while the nodules grow larger and can reach approximately the size of a walnut. The adipocytes become hypertrophic and the fibrotic areas enlarge in the affected regions. An increased recruitment of anti-inflammatory macrophages is observed, but the number of pro-inflammatory macrophages also increases (Figure 2). Further, thickening of capillary vessels and hyperplasia of adipogenic stem and progenitor cells (ASPCs) has been described (Priglinger et al., 2017; Kruppa et al., 2023; Michelini et al., 2025; Al-Ghadban et al., 2026). In the final stage, the nodules continue to grow and harden, leading to pronounced deformation of the affected limbs and joints. The disease-affected regions of SAT show clear signs of inflammation, with increased numbers of pro- and anti-inflammatory macrophages (Figure 2). The tissue is markedly fibrotic and features thick and leaky vessels, leading to interstitial fluid accumulation (Allen et al., 2020; Allerton, 2025).

**TABLE 1:** Summary of representative study details analyzing ASPCs in SAT of patients with lipedema.

Study	Lipedema stage	Cohort size	Age	BMI (kg/m^2^)	Sample type	Isolation method	Sample handling and culture conditions	Growth medium	Cell numbers	Read out
Al Ghadban 2019	SI - III	C: 10 non-obese C: 9 obese L: 16 non-obese L: 15 obese	C: 40.6±4.2 non-obese C: 43.3±5.2 obese L: 43.3±2.5 non-obese L: 46.1±2.4 obese	C: 23.5±0.7 non-obese C: 34.1±1.4 obese L: 25.2±0.5 non-obese L: 34.1±0.8 obese	Punch Biopsies (5mm)	no digest, full tissue	Paraffin embedding	---	---	Histology, Immunohistochemistry, Immunofluorescence
Al Ghadban 2020	SI - III	20 per group SI: 15% SII: 70% SII-III: 5% SIII: 10%	C: 46.9±2.2 L: 44.1±2.2	C: 28.5±0.86 L: 29.9±0.97	Lipoaspirate	Col 1 0.1%	Passage 3, 1D culture, Adipogenic differentiation 21 days	DMEM/F12, 10% FCS	2x10^4^ cells/cm^2^	Gene expression analysis, Cell growth and differentiation assays
Al Ghadban 2020	SI - III	8 per group SI: 12% SII: 63% SIII: 25%	C: 47.86±2.28 L: 44.57±3.99	C: 27.3±1.04 L: 30.43±1.16	Lipoaspirate	Col 1 0.1%	Passage 3, spheroid formation, Adipogenic differentiation 10 days	DMEM/F12, 10% FCS	30x10^3^/cm^2^ in 12 well	Gene expression analysis, Cell differentiation assays, Immunofluorescence
Bauer 2019	SII	10 per group	≤ 18 years	not mentioned	Lipoaspirate	Col A 0.1%	Passage 3-4, Adipogenic differentiation 7 or 10 days	Stem MACS MSC expansion medium	2000 cells/ 6 well	Cell growth and differentiation assays
Ernst 2024	not mentioned	C: 6 L NW: 10 L OB: 5	C: 33.4±8.4 L NW: 31±6.9 L OB:29.4±1.5	C: 25.4±2.1 L NW: 24.4±2.3 L OB: 36.2±4.6	Lipoaspirate	Col A 0.1%	Passage 0-3 Adipogenic differentiation for 21 days	DMEM high glucose, 10% FCS	not mentioned for 2D, 6000 cells / drop for 3D	Gene expression analysis
Felmerer 2020	SI - III	C: 11 L: 10	not mentioned	C: 28.39±3.93 L: 27.16±2.19	Biopsies from skin and fat tissue	no digest, full tissue	Fresh frozen tissue / Paraffin embedding	---	---	Histology, Immunohistochemistry, Gene expression analysis
Ishaq 2022	SII and SIII	C:10 L:14	not mentioned	C and L: 26-36	Biopsies (10g)	Col 1 1mg/ml	Passage 3-5	DMEM high glucose, 10% FCS	40000 cells/cm^2^	RNA-seq Flow Cytometry, Cell culture
Kruppa 2023	SI - SIII stage dependent analysis	C: 14 SI: 9 SII: 16 SIII: 7	C:38.71±9.9 SI: 31.56±8.9 SII: 37.31±12.9 SIII: 45.3±12.2	C: 26.7±4.8 SI: 25.1±3.3 SII: 29.9±3.03 SIII: 31.4±3.7	Biopsies (subcutaneous and deep tissue)	no digest, full tissue	Fresh frozen tissue / Paraffin embedding	---	---	Histology, Immunofluorescence Gene expression analysis
Pagani 2024	not mentioned	C: 15 L: 15	18-45 years	< 35	Punch Biopsies from subcutaneous tissue layer and from deep layer under the Scarpa´s fascia (6 mm)	Col 2, no concentration mentioned	Freshly isolated cells, directly frozen after digestion	---	---	Single cell RNA-seq
Priglinger 2017	SII and SIII	C: 22 L: 30 SII 55% SII-III 24% SIII 21%	C: 42±10 L: 41±13	C: 27±6 L: 33±7	Lipoaspirate and Biopsies	Col NB4 0.2 U/ml	Passage 0 - 3 / Paraffin embedding	EGM-2	1.4 x 10^4^ / 24 well for adipogenic differentiation	Flow Cytometry, Cell differentiation assays, Histology
Streubel 2024	SI - III	C: 8 L: 54 SI: 24 SII: 17 SIII: 13	C: 42.7±14.2 SI: 39.9±8.9 SII: 43±10.7 SIII: 48.2±11.2	C: 25.98±3.6 SI: 29.8±5.3 SII: 31.92±4.65 SIII: 36.93±6.25	Forceps Biopsies	no digest, full tissue	Freshly isolated RNA	---	---	RNA-seq
Strohmeier 2022	SI and SII	C: 4 L: 5	C: 37.8±2.2 L: 33.6±10.7	C: 23.3±3 L: 29.5±2.5	Lipoaspirate and Punch Biopsies (4mm) with dermis	Col NB4 0.1U/ml	Freshly isolated cells or cultured to passage 2 / Paraffin embedding	EGM-2	1 x 10^6^ or 1 x 10^5^ / T-25 flask	Flow Cytometry, Gene expression analysis, Histology
Wolf 2022	SI - SIII	C: 19 L: 20	C: 50.17± 12.36 L: 48.32±12.69	C: 26.81±4.43 L: 28.6±3.95	Biopsies from skin and fat tissue	Col 2 2 mg/ml	Fresh frozen tissue / Paraffin embedding, freshly isolated cells, directly frozen after digestion	---	---	Histology, Gene expression analysis, CyTOF, RNA-seq

Comparison of representative study details analyzing gene and protein expression in SAT of patients with lipedema (abbreviated with L) in comparison to the healthy comparator group (abbreviated with C). The distribution of patients in different lipedema stages from stage I to stage III (abbreviated with SI, SII, SIII) was included if applicable. Further cohort size, age and BMI, sample type, cell isolation method regarding the collagenase type used (abbreviated with Col) and concentration if applicable, culture/treatment conditions, growth medium and cell numbers if applicable, as well as read out method. In some studies, lipedema donors were separated in normal weight (L NW) and obese (L OB) (Priglinger et al., 2017; Al-Ghadban et al., 2019; Bauer et al., 2019; Al-Ghadban et al., 2020a; Al-Ghadban et al., 2020b; Felmerer et al., 2020a; Ishaq et al., 2022; Strohmeier et al., 2022; Kruppa et al., 2023; Ernst et al., 2024; Pagani et al., 2024); Created in BioRender. Gohlke, S. (2026) https://BioRender.com/7go6ujg.

**FIGURE 2:**
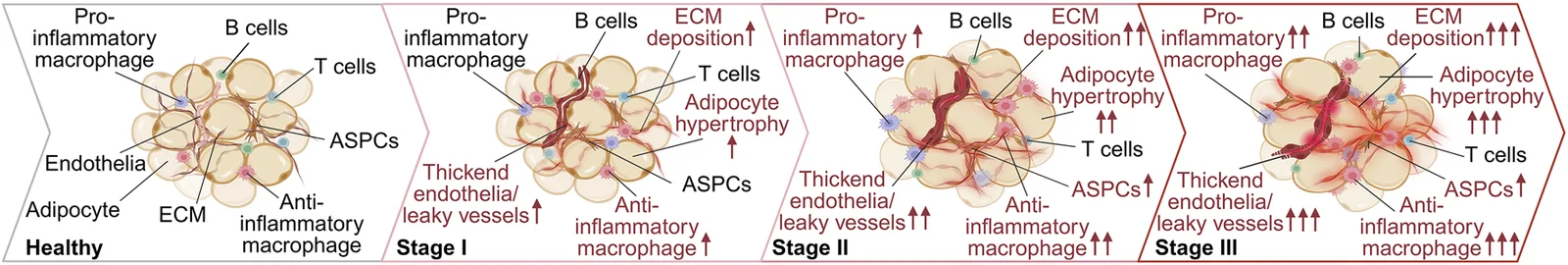
Stage dependent alterations of lipedema affected tissue composition. SAT of healthy normal weight individuals is composed of adipogenic stem and progenitor cells (ASPCs), mature adipocytes, endothelia and immune cells like pro- and anti-inflammatory macrophages as well as T- and B cells that are connected by extracellular matrix (ECM; left panel, grey line). In patients with lipedema, the tissue structure of SAT in the thigh undergoes progressive changes that intensify with increasing stages: In stage I (middle left panel, light pink line) ECM deposition, thickened endothelia and leaky vessels are detectable. Further, higher proportions of macrophages of the anti-inflammatory type 2 accumulate in lipedema affected tissue compared to healthy individuals. In stage II (middle right panel, pink line), mature adipocytes appear hypertrophic, ECM deposition, the endothelia and vessel phenotype worsen. Higher numbers of ASPCs were measured in lipedema affected tissue compared to healthy donors. In addition to accumulation of anti-inflammatory macrophages type 2, recruitment of pro-inflammatory macrophages type 1 was described. In stage III (right panel, red line), significant tissue remodeling is evident based on a high fibrotic index indicating matrix remodeling, increased recruitment of pro- and anti-inflammatory macrophages and significant changes in endothelial and vascular integrity. Adipocytes are hypertrophic and high number of progenitors were measured (Priglinger et al., 2017; Allen et al., 2020; Strohmeier et al., 2022; Kruppa et al., 2023; Allerton, 2025; Michelini et al., 2025; Al-Ghadban et al., 2026). Created in BioRender. Gohlke, S. (2026) https://BioRender.com/z00ird3.

Secondly, as another potential source of discrepant observations in lipedema research, technical variances between studies can cause significant differences in outcomes (Table 1). The type of tissue sampling, e.g., punch biopsy or lipoaspirate, is important to consider, as molecular signatures are different between superficial and deep sites of SAT (Cancello et al., 2013; Dam et al., 2026). Another factor causing potentially contradictory results might be the *in vitro* handling of isolated adipogenic progenitor cells (Table 1), as gene expression profiles and proliferation capacities of these cells can be affected by parameters like seeding density, composition of culture media and specifics of the differentiation protocol (Bunnell et al., 2008; Roxburgh et al., 2016). In order to reliably compare study results, it is therefore essential to factor in such differences in study design and to harmonize guidelines on diagnosis, analysis and reporting procedures, ranging from lipedema sample collection to cell biology investigations.

## The molecular identities of mature adipocytes in lipedema

Adipose tissue is composed of different types of cells whose proportions vary substantially between different depots. Mature adipocytes represent the bulk of WAT volume, expressing marker genes such as *ADIPOQ* and perilipin 1 (*PLIN1*). Adipocytes are responsible for energy storage as triglycerides and characterized by a large central lipid droplet that covers around 90% of the cells’ volume. In studies with healthy volunteers, it was shown that femoral subcutaneous adipocytes have higher insulin sensitivity and enhanced capacity for triglyceride storage with reduced release of non-esterified fatty acids (NEFA) after metabolic stimuli, when compared to mature subcutaneous abdominal adipocytes (McQuaid et al., 2010). Further, it was shown that an increase of visceral adipose tissue (VAT), even when simultaneously reducing SAT, exacerbates insulin resistance in individuals with type 2 diabetes (Gallagher et al., 2009). This might also correspond to the observation of improved blood lipid profiles in patients with lipedema that might be due to the disproportional accumulation of SAT with high capacity for triglyceride storage (Koster et al., 2010; Pinnick et al., 2014). However, certain metabolic features of the affected adipocytes seem to be impaired in patients with lipedema, as these adipocytes become largely resistant to dietary interventions and exercise effects (Torre et al., 2018).

Using single nucleus RNA-sequencing (snRNA-seq) of human SAT and VAT, distinct clusters of adipocytes were described by Emont et al. in a study unrelated to lipedema. Specifically, four distinct BMI-correlated adipocyte clusters, termed hAd1, hAd3, hAd4 and hAd7, were linked to SAT by unique marker expression patterns and functions (Emont et al., 2022). For instance, hAd1 represented up to 40% of adipocytes in SAT, but its marker gene expression profile was relatively unspecific and therefore was described as the “basal” or “classical” adipocytes. Contrasting this, more specific SAT-derived clusters hAd3, expressing high levels of the adipokines *ADIPOQ* and complement factor D (*CFD;* also: adipsin)*,* and hAd4, marked by expression of glutamate ionotropic receptor AMPA type subunit 4 *(GRIA4*), were also identified. Both clusters were additionally enriched in genes associated to lipogenesis, triglyceride biosynthesis, and fatty acid desaturation. While hAd4 and hAd7-proportions negatively correlated to BMI, hAd5 proportion was positively correlated to BMI. The BMI-dependent dynamics in SAT-derived adipocytes were also confirmed in another study that reported a strong dependence of adipocyte plasticity on metabolic health (Reinisch et al., 2025). Specifically, it was shown that adipocytes which functionally clustered to growth-related pathways and mitogen activated protein kinase (*MAPK*) signaling were strongly enriched in SAT of patients suffering from metabolically unhealthy obesity (Reinisch et al., 2025). This cluster, marked by expression of neuronal-related cell adhesion molecule (*NRCAM*), resembled cluster hAd4 described by Emont et al. (Emont et al., 2022).

An unresolved question remains how adipocyte heterogeneity is affected by lipedema. While it was reported that adipocytes are hypertrophic in lipedema-affected adipose tissue, it remains to be elucidated whether transcriptomic signature changes occur in the adipocyte fraction itself, leading to shifts in existing cluster proportions, or formation of other distinct, novel adipocyte clusters in adipose tissue affected by lipedema. Currently, only one study has used single cell RNA sequencing (scRNA-seq) to analyze adipocyte populations in lipedema affected SAT (Pagani et al., 2024). Here, adipose tissue samples of healthy individuals and patients with lipedema were compared following collagenase digestion of the samples. The stromal-vascular fraction (SVF) was collected together with the floating layer of adipocytes and used for scRNA-seq. The authors reported three different adipocyte clusters in their study. Cluster ‘adipocyte A’ expressed markers like apolipoprotein D (*APOD*)*,* matrix-Gla-protein (*MGP*)*,* insulin-like growth factor 1 (*IGF1*), aldehyde dehydrogenase 1 family member A1 (*ALDH1A1*)*,* and C-X-C motif chemokine ligand 14 (*CXCL14*), linking it to support of adipogenesis and lipid transport, while cluster ‘adipocyte B’ expressed complement decay-accelerated factor (DAF; also: *CD55*)*,* semaphorin 3C (*SEMA3C*)*,* versican (*VCAN*)*,* and secreted frizzled-related protein 4 (*SFRP4*) which link this cluster to lymphatic destabilization and lipid storage. Both clusters were found in lipedema and healthy adipose tissue according to the characteristic distribution profile seen in the disease. The ‘adipocyte C’ cluster, characterized by expression of fatty acid binding protein 4 (*FABP4*)*,* cluster of differentiation 36 (*CD36*)*,* retinol binding protein 7 (*RBP7*)*,* serum deprivation response (*SDPR*)*,* alpha-2-makroglobulin (*A2M*) and interferon alpha inducible protein 27 (*IFNI27*) markers, was presented as a group of hypertrophic, lipedema-specific adipocytes and the formation was described to be facilitated by both ‘adipocytes A and B’ clusters (Pagani et al., 2024).

Although the overlap of genes defining different clusters of adipocytes and progenitors between different studies is relatively low, the ‘adipocyte A’ marker expression profile to some extent resembles a progenitor cluster, hASPC1, described in the study by Emont et al., which also features expression of genes *APOD*, *MGP*, *IGF1* and *CXCL14* among the top markers (Emont et al., 2022). The ‘adipocyte A’ cluster further resembled a cluster of committed preadipocytes in the late stages of differentiation reported by Massier et al., as both clusters show enriched expression of marker genes *CXCL14*, *APOD* and *CFD* (Massier et al., 2023). These observations may support the assumption that the low overlap of molecular signatures between different studies might at least in part be due to technical and biological differences such as using snRNA-seq and scRNA-seq, bioinformatic processing and differences in inclusion criteria, which altogether may contribute to discrepant identifications and characterizations of cell clusters. This hypothesis is further supported by the observation that the ‘adipocyte A’ cluster from Pagani et al. could also be functionally similar to the majority of classical adipocytes denoted as a cluster of subcutaneous adipocytes, termed SA1, by Lazarescu et al. or the mature adipocyte clusters hAd1 and hAd2 defined by Emont et al., which were enriched for fatty acid and lipid metabolism-associated terms (Emont et al., 2022; Lazarescu et al., 2025). ‘Adipocyte B’ displayed several similar markers as Emont et al.’s progenitor cell cluster, hASPC2, which is marked by expression of *SEMA3C*, *CD55*, *SFRP4* and *VCAN*, and which resembled early multipotent progenitors that are located in the reticular interstitium and therefore would be involved in lymphatic stabilization (Emont et al., 2022), but could also be described as non-classical adipocyte clusters termed SA4 or hAd4 by Lazarescu et al. and Emont et al., respectively, that are enriched for lymphocytic processes including T cell activation and adaptive immune responses (Emont et al., 2022; Lazarescu et al., 2025). The ‘adipocyte C’ population described by Pagani et al. resembled none of the described adipocyte clusters for the studies on healthy or obese human WAT when considering overlapping expression of single marker genes, although *FABP4* and *CD36* are described as typical adipocyte markers regulating lipid metabolism and play a role in the development of inflammation (Terra et al., 2011; Massier et al., 2023).

Given the limited volume of currently available data, it is not yet possible to determine lipedema-related effects on mature adipocyte and ASPC heterogeneities on the single cell or even sub-population level. As some of the marker genes used to define the populations in this study are also described as markers of developing adipocytes (Albert et al., 2026), it needs to be evaluated more conclusively whether the adipocyte populations are indeed mature adipocytes with lipedema-specificity, or whether they differ in their differentiation stage. In general, the substantial methodological differences between single cell-based studies and approaches to define mature adipocyte sub-populations result in an overlap that is relatively limited, which might be due to the fact that adipocytes are spatially organized in the tissue within discrete functional niches, where cell-cell interactions are important for cellular functions (Backdahl et al., 2021). A resolution pertaining to the remaining open questions on lipedema-related changes in adipose tissue might be accomplished through spatial transcriptomics technologies, where genes are mapped to intact tissue sections and therefore could provide an overview about compositions of potentially distinct adipogenic niches, cell-cell interactions, and immune cell infiltrations.

## Lipedema-associated dysregulation of adipogenic stem and progenitor cell development

Most of the studies on adipogenic progenitor cells conducted to date are limited to the analysis of in vitro-differentiated cells, which are isolated from SAT biopsies of healthy donors and patients with lipedema, followed by enzymatic digestion. These cells are defined as the SVF, and such cell cultures are typically depleted of immune, endothelial and red blood cells (Rodeheffer et al., 2008; Sanchez-Gurmaches and Guertin, 2014). Since these cell isolates are highly heterogenous, they are commonly summarized by the term adipogenic stem and progenitor cells (ASPCs); (Ferrero et al., 2020). They are likely comparable to the terminology of tissue-resident, non-hematopoietic mesenchymal stromal cells (MSCs) that can give rise to osteocytes, adipocytes and chondrocytes due to their multipotency. Generally, it is agreed that these cells are marked by expression of endoglin (*ENG;* also: *CD105*)*,* ecto-5'-nucleotidase (*NT5E;* also: *CD73*)*, CD34* and the Thy-1 cell surface antigen (*Thy1;* also: *CD90*); (Pittenger et al., 1999; Dominici et al., 2006), but recent studies summarizing ASPC features postulate that only subsets of ASPCs are recognized by these markers (Albert et al., 2026). Progenitor subsets committed to the adipogenic lineage are additionally marked by expression of *PPARG*, which in turn regulates the expression of platelet derived growth factor receptor alpha (*PDGFRA*) and *C/EBPα*. Adipogenesis is tightly regulated by different transcription factors, whose activity is regulated by secreted molecules of the canonical Wingless-related integration site (WNT) signaling pathway, showing depot-specific properties. In this line, SAT-derived progenitors accumulate more lipids during adipogenic differentiation, have higher proliferation rates and express higher levels of typical adipogenic marker genes like *PPARG, FABP4* and *C/EBPα* compared to VAT-derived progenitors (Perrini et al., 2008).

Various studies agree that ASPCs can be subdivided into subpopulations with different properties by surface marker expression profiles (Merrick et al., 2019; Ferrero et al., 2020; Emont et al., 2022; Divoux et al., 2024; Wang et al., 2024; Lazarescu et al., 2025; Reinisch et al., 2025; Whytock et al., 2026). High resolution studies using sn- or sc-RNA-seq analyses of human adipose tissue confirm this observation and have revealed a high level of heterogeneity, with several subclusters of ASPCs representing cells at different stages of differentiation (Table 2). Among these, early-stage uncommitted interstitial progenitors (UMPs) show a high degree of stemness and are marked by expression of dipeptidyl-peptidase 4 (*DPP4;* also: *CD26*), peptidase inhibitor 16 (*PI16*) and *CD55* and have high proliferation capacity (Merrick et al., 2019; Ferrero et al., 2020; Wang et al., 2024). The UMP subset in turn gives rise to uncommitted, intermediate preadipocytes (IPAs) which express intercellular adhesion molecule 1 (*ICAM1*)*,* integrin subunit beta 1 (*ITGB1*)*,* CD44 molecule (*CD44*) and coagulation factor III (*F3;* also: *CD142*); (Merrick et al., 2019; Lazarescu et al., 2025; Albert et al., 2026). The commitment from IPAs towards the adipogenic lineage, forming intermediate preadipocytes (CPA), is marked by increased expression of *PPARG,* zinc finger protein-423 (*ZNF423*)*,* delta-like non-canonical notch ligand 1 (*DLK1*)*,* ADP-ribosyl cyclase 1 (*ADPRC1;* also: *CD38*) and gamma-glutamyltransferase *5* (*GGT5*); (Massier et al., 2023; Divoux et al., 2024; Wang et al., 2024; Reinisch et al., 2025). During adipogenesis, these cells are able to form premature adipocytes (PreAds) expressing, among other genes, *PPARG, CXCL14, APOD, CD36, MGP, CFD* and *CEBPB* (Ferrero et al., 2020; Emont et al., 2022; Gu et al., 2023; Whytock et al., 2026). In the end, mature adipocytes are formed, which express well-known mature adipocyte marker genes like *ADIPOQ*, *PLIN1* and *LEP* (Emont et al., 2022; Lazarescu et al., 2025). Additionally, non-adipogenic subtypes of ASPCs were described that could arise from the IPA subset upon metabolic stress, hypoxia or infections and exhibit profibrotic or proinflammatory properties. These populations were, among others, described by expression of *F3*, ephrin type-A receptor A3 (*EPHA3*)*,* potassium voltage-gated channel interacting protein 1 (*KCNIP1*) and insulin-like growth factor binding protein 7 (*IGFBP7*), a cluster representing the anti-adipogenic adipogenesis regulatory cells (Aregs), and a cluster with profibrotic progenitors expressing tetraspanin (*CD9*), versican (*VCAN*), lymphocyte antigen six family member C1 (*Ly6C*)*,* collagen 14 A1 (*COL14A1*)*,* disintegrin and metalloproteinase domain-containing protein 12 (*ADAM12*) and periostin (*POSTN*)*,* (Schwalie et al., 2018; Ferrero et al., 2020; Emont et al., 2022; Massier et al., 2023; Wang et al., 2024; Reinisch et al., 2025).

**TABLE 2:** Cellular composition of subcutaneous adipose tissue.

**Cell type**	**Population**	**Feature**	**Marker**	**Lipedema dependent regulation**	**Reference**
ASPCs	Mesenchymal stem cells (MSC)	Multi lineage potential	CD105, CD73, **CD34** **,** **CD90**	**Upregulated?**	Priglinger et al. (2017), Dominici et al. (2006), Suga et al. (2009)
	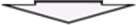 Early uncommitted multipotent progenitors (UMP)	Early ASPCs, adipogenic potential, reside in reticular interstitium	DPP4, CD55, Pl16, Aldh1A1, Aldh1A3	Not reported	Massier et al. (2023), Emont (2025), Merrick (2019)
	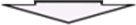 Uncommitted, intermediate preadipocytes (IPA)	Pre-commitment into the adipogenic lineage, intercalated between mature adipocytes	ITGB1, CD44, ICAM1	Not reported	Massier et al. (2023), Emont (2025), Merrick (2019)
	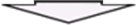 Committed, intermediate preadipocytes (CPA)	Pre-adipocyte population, located in the neurovascular niche, but no neurons or endothelial cells	**ZNF423** , DLK1, CD38, GGT5	**Upregulated?**	Whytock, (2026), Massier (2023), Strohmeier (2022)
	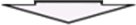 Premature adipocyte (PreAd)	Committed preadipocytes	**PPARG** , APOD, **CFD** , CD36, APOE, MGP, GPX3, C/EBPB	**Upregulated!** **Downregulated?**	Massier (2023), Felmerer et al. (2020a)
Mature adipocytes	HAD1/SA1	Basal adipocytes found in SAT	ADIPOQ, PLIN1, **LEP** , AQP7, LDL	Not reported	Emont et al. (2022), Lazarescu et al. (2025)
	HAD4/SA4	Immune modulating adipocytes found in SAT	ADIPOQ, PLIN1, ELOVL5, FADS3, ABCD2, STAT4, GRIA4	Not reported	Emont et al. (2022), Lazarescu et al. (2025)
	HAD5/SA5	Extracellular matrix remodeling adipocytes found in SAT	ADIPOQ, INSR, IRS2, PDE3B, ACER2, FYN, SPTLC3, PGAP1	Not reported	Emont et al. (2022), Lazarescu et al. (2025)
	HAD7/SA7	Not specified, maybe plays a role in development of T2D	ADIPOQ, **CFD** , AGMO	Not reported	Emont et al. (2022), Lazarescu et al. (2025)
	Adipocyte A	Adipogenesis, lipid transport	ApoD, MGP, IGF, ALDH1A1, CXCL14	**Upregulated**	Pagani et al. (2024)
	Adipocyte B	Lymphatic destabilization, cell accumulation	CD55, SEMA3C, VCAN, SFRP4	Not affected	Pagani et al. (2024)
	Adipocyte C	Lipidemic adipocytes, hypertrophic	FABP4, CD36, RBP7, SDPR, A2M, IFNI27	**Upregulated**	Pagani et al. (2024)
Non adipogenic progenitors	Pericyte progenitor cells	Endothelia/pericyte subpopulation (arise from IPAs)	**CD146** , CD29	**Upregulated?**	Priglinger et al. (2017)
	Fibro adipogenic progenitors (FAPS)	Pro fibrotic cells, no adipogenic potential, proinflammatory mediators (arise from UMP, IPA, CPA)	PDGFRA, CD9, **CD34** , VCAN	Not reported	Massier et al. (2023), Emont et al. (2022), Albert et al. (2026), Lazarescu et al. (2025)
	Anti adipogenic regulatory cells (Aregs)	Anti adipogenic, regulate angiogenesis and immune response (arise from UMP, IPA, CPA)	CD142, EPHA3, KCNIP1, IGFBP7	Not reported	Emont et al. (2022), Whytock et al. (2026), Reinisch et al. (2025)
	Endothelia cells	Lining interior of blood and lymphatic vessels	CD31, **VEGF-C/** **VEGFR-3**	Not affected/ **Increased systemically**	Strohmeier et al. (2022), Felmerer et al. (2020), Priglinger et al. (2017)
Immune cells	Immune cells	Nucleated cells of hematopoietic origin	**CD45**	Not affected**/ Upregulated**	Cifarelli et al. (2025), Felmerer et al. (2020b)
	T- cells	Cells of the adaptive immune response	CD3, CD4, CD8	Not affected/ Not reported	Felmerer et al. (2020a)
	Mast cells	Rapid immune response, tissue resident located at fibrotic bundles, perivascular and dispersed between adipocytes	**Histamine**	**Upregulated**	Bonetti et al. (2023)
	Macrophages	Macrophage/monocyte immune cells central to immune defense, tissue repair and inflammation	**CD11c, CD68**	**Not affected/ Upregulated**	Wolf et al. (2022), Strohmeier et al. (2022), Sugaet al. (2009), Al-Ghadban et al. (2019), Felmerer et al. (2020a)
	Pro-inflammatory macrophages (M1)	Pro-inflammatory effector cells involved in chronic inflammation and tissue damage	**CD86**	**Upregulated in stage 3**	Kruppa et al. (2023)
	Anti-inflammatory macrophages (M2)	Anti-inflammatory and tissue repairing functions. essential for tissue homeostasis	**CD163, CD206**	**Upregulated**	Streubel et al. (2024), Kruppa et al. (2023), Wolf et al. (2022)

Summary about cell populations identified in subcutaneous adipose tissue with features and typical markers. Markers that were reported to be affected by lipedema are highlighted in bold red. The lipedema dependent regulation is listed based on published studies cited in the last column and hypothetical lipedema dependent regulation is listed in italic letters. ASPC subpopulations (light green) with arrowheads indicating the developmental hierarchy, forming in the end mature adipocytes (light red). Here, different subtypes of mature adipocytes were described for SAT in different studies. Additionally, non-adipogenic progenitors (green) and immune cells (blue) were identified in SAT, where the most prominent changes about cellular composition were reported for endothelial cells and macrophages (Dominici et al., 2006; Suga et al., 2009; Priglinger et al., 2017; Al-Ghadban et al., 2019; Merrick et al., 2019; Felmerer et al., 2020a; Felmerer et al., 2020b; Emont et al., 2022; Strohmeier et al., 2022; Wolf et al., 2022; Bonetti et al., 2023; Kruppa et al., 2023; Massier et al., 2023; Pagani et al., 2024; Streubel et al., 2024; Cifarelli et al., 2025; Lazarescu et al., 2025; Reinisch et al., 2025; Albert et al., 2026; Whytock et al., 2026). Created in BioRender. Gohlke, S. (2026) https://BioRender.com/0g6nu6e.

Heterogeneity of ASPCs is depot-specific and can vary substantially under different metabolic states (Emont et al., 2022; Reinisch et al., 2025; Whytock et al., 2026). Due to the high plasticity of adipogenic progenitors and the variability of marker gene expression, it is difficult to describe lipedema-related effects on ASPCs and the number of available studies is limited. Proliferation and differentiation capacities were analyzed under different experimental conditions, for instance by seeding the SVF in regular culture dishes (Priglinger et al., 2017; Bauer et al., 2019; Al-Ghadban et al., 2020a; Ishaq et al., 2022; Strohmeier et al., 2022) or by seeding cells in three-dimensional cell culture systems (Al-Ghadban et al., 2020b). Typical adipogenic marker expression like *PPARG, LEP, ADIPOQ,* lipoprotein lipase (*LPL*)*, CD36* or solute carrier family 2 member 4 (*SLC2A4*; also: *GLUT4*) were investigated after variable times of differentiation (Table 1). When comparing progenitors isolated from adipose tissue biopsies of patients with lipedema to healthy donor cells, contradictory phenotypes have been reported: the cells isolated from lipedema affected tissue have higher adipogenic potential marked by increased expression of *PPARG* and *LEP* (Al-Ghadban et al., 2020a), no changes in adipogenic potential (Al-Ghadban et al., 2020b), or even decreased adipogenic potential, marked by downregulation of *CEBP/d* (Felmerer et al., 2020a) and lower concentrations of LEP and ADIPOQ in the culture supernatant (Bauer et al., 2019). The differences between the gene expression patterns in these studies may not only arise due to different culture conditions and differentiation time courses (Priglinger et al., 2017; Ernst et al., 2023), but also due to donor cohort composition (Table 1). It is therefore important to take into account that technical variables, such as tissue sample collection style, tissue digestion conditions during the cell isolation process, cell seeding density and cultivation period, as well as media compositions are critical to differentiation outcomes.

The effect of lipedema on the cellular composition of freshly isolated ASPCs was investigated by analysis of the surface marker profiles using flow cytometry (Tables 1 and 2; Figure 3). ASPCs isolated from lipedema-affected tissue showed a higher proportion of cells positive for surface marker protein CD90 and an increase of pericyte surface marker melanoma cell adhesion molecule (MCAM, also: CD146) compared to healthy individuals (Priglinger et al., 2017). Importantly, this difference was lost after 1 week of *in vitro* cultivation, again highlighting the importance of considering the experimental conditions used (Priglinger et al., 2017). To reliably describe ASPC trajectories, it may therefore be necessary to analyze the cellular compositions immediately after tissue collection and cell isolation, while prolonged cell culturing and passaging of cells should be avoided as this could introduce altered and non-physiological surface marker profiles. Furthermore, higher proportions of CD29-and CD34-presenting ASPCs were observed in adipose tissue samples affected by lipedema, indicating changes in cellular composition and potentially its microenvironment (Ishaq et al., 2022). This was driven by altered expression of the gene mitotic checkpoint serine/threonine kinase (*BUB1*), a kinase known to control cell cycle and cell growth (Ishaq et al., 2022).

**FIGURE 3:**
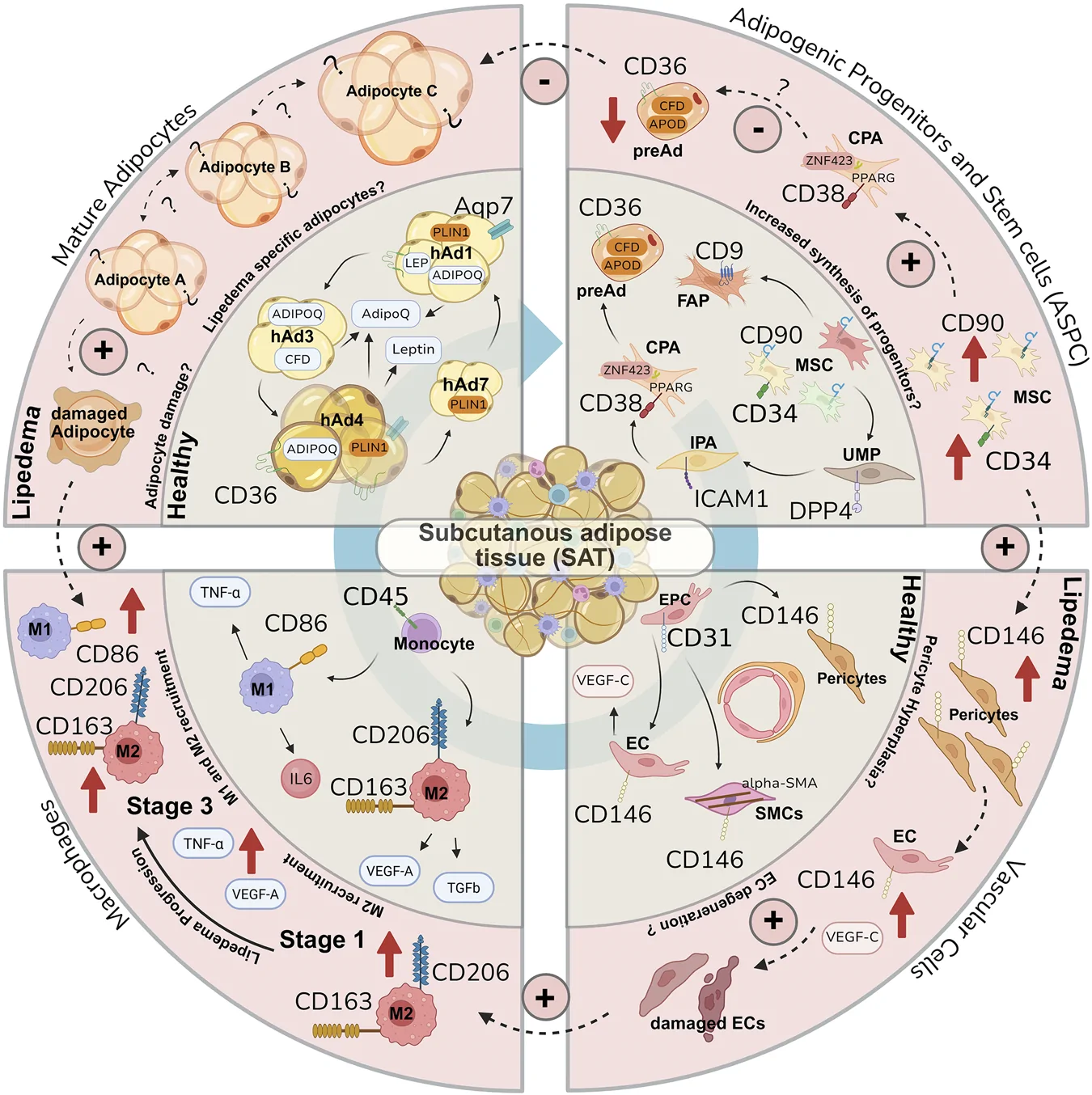
Surface marker composition of main cell types in lipedema affected SAT. Lipedema-affected changes of cellular composition and marker gene expression might affect cellular interaction dynamics (connections are indicated by dashed arrows): Top left, mature adipocytes: Healthy adipose tissue (green area) contains multiple adipocyte subclusters (hAd1, hAd3, hAd4 and hAd7) characterized by defined gene expression patterns (e.g., *ADIPOQ, PLIN1, LEP, CFD*) that secrete typical adipokines like LEP, ADIPOQ and CFD. The effect of lipedema on mature adipocytes remains to be elucidated, but it might lead to the formation of lipedema-specific adipocyte populations with uncertain origin and function (red area, indicated by question marks). Top right, adipogenic stem and progenitor cells (ASPCs): Healthy SAT (green area) contains uncommitted mesenchymal stromal cells (MSCs) characterized by, among others, *CD90* and *CD34* gene expression. They give rise to uncommitted interstitial progenitors (UMPs, *DPP4* gene expression), intermediate preadipocytes (IPAs, *ICAM1* gene expression) and committed, intermediate preadipocytes (CPAs, *ZNF423*, *CD38*, *PPARG* gene expression) that develop in the next step to premature adipocytes (preAds, *CD36*, *CFD*, *APOD* gene expression). In parallel, non-adipogenic subsets (fibro adipogenic progenitors, FAPs) expressing *CD9* are detected. Lipedema (red area), is associated with an increased expression of *CD90 and ZFP423*, which could indicate an increased production of MSCs and, consequently, increased formation of CPAs. In contradiction to this, due to the reduction of *CFD* expression it can be assumed that the formation of preadipocytes is disturbed in lipedema, paralleled by higher formation of pericytes. Bottom right, vascular cells: In healthy state (green area), endothelia progenitor cells (EPCs, *CD31* *+*) give rise to ECs (*CD146+*) and smooth muscle cells (SMCs, *CD146+*, *α-SMA+*) forming and stabilizing vessels together with pericytes (*CD146+*). The pathology of lipedema (red area) linked to higher gene expression of *CD146* indicating EC and/or pericyte hyperplasia. It is likely that dysfunctional cells are formed, which cause leakage and instability in the vessels and stromal inflammation with increased secretion of VEGF-C. Bottom left, macrophages: In healthy adipose tissue (green area), monocytes mainly differentiate into anti-inflammatory macrophages type 2 (M2, *CD163+, CD206+*), while a minor subset of proinflammatory macrophages type 1 (M1, *CD86* *+*) resides in the tissue. The early lipedema stage I is characterized by high recruitment of M2. In lipedema stage III, a chronic inflammatory milieu develops due to accumulation of M1 and M2 macrophages reflected by higher secretion of cytokines like TNFα and VEGF (red area). (Priglinger et al., 2017; Felmerer et al., 2020b; Emont et al., 2022; Ishaq et al., 2022; Strohmeier et al., 2022; Kruppa et al., 2023; Pagani et al., 2024; Michelini et al., 2025). Created in BioRender. Gohlke, S. (2026) https://BioRender.com/06cwv2d.

Taken together, the published evidence suggest that lipedema leads to increased accumulation of ASPCs in affected regions of SAT, which are characterized by surface expression of CD90. This could lead to an increased formation of *ZNF423*-expressing adipogenic progenitor cells, which in contrast to healthy adipose tissue, do not progress into adipocytes in patients with lipedema, but rather result in an increased accumulation of perivascular cells that express the surface marker CD146. This could in turn contribute to formation of an extracellular environment that promotes a dysfunction of the endothelial barrier.

## Endothelial dysfunction and pericyte remodeling in lipedema

ECs form the inner layer of blood vessels and are stabilized by an outer layer of pericytes, thereby supporting vessel maturation and regulating nutrient exchange with a tissues’ parenchymal cells, i.e., adipocytes within adipose tissue. In addition, the adipose tissue endothelium controls lipid storage in adipocytes through the regulation of lipoprotein lipase activity and fatty acid transport (Davies et al., 2012). Various fatty acid transporters are expressed in ECs, with *CD36* being a prominent representative. Furthermore, they promote angiogenesis in situations of tissue expansion and enable bidirectional signaling crosstalk with adipocytes, therefore promoting adipocyte differentiation through the expression of *PPARG* cofactors (Gogg et al., 2019). In obesity, dysfunctional ECs can promote chronic inflammation and result in disturbed angiogenesis, which can be assessed by expression of vascular endothelial growth factor (*VEGF*); (Gogg et al., 2019). In lipedema, increased permeability of microvascular structures and elevated vessel density was observed, leading to an excessive fluid accumulation in the interstitial space, causing hypoxia, edema and microangiopathy in the affected tissue (Al-Ghadban et al., 2019; Allen et al., 2020). Remarkably, this was only observed in lipedema-affected tissue in the extremities, but not in abdominal regions, which presents a lipedema-specific phenotype that us distinct from obesity. Accordingly, increased circulating levels of VEGF-C were measured in plasma of patients with lipedema compared to healthy donors that might lead to the formation of immature, hyperpermeable blood vessels (Al-Ghadban et al., 2019; Felmerer et al., 2020b). Expression analyses further revealed that the markers for lymphatic and blood vessels, tyrosine kinase with Ig and EGF homology domains-2 (*TIE2*) and fms-related receptor tyrosine kinase 4 (*FLT4,* also: *VEGFR3*), were downregulated, accompanied by locally affected cytokine milieu indicating increased vascular permeability (Felmerer et al., 2020b). Accordingly, by analyzing high resolution images using transmission electron microscopy, Michelini et al. showed capillary wall thickening and endothelial degeneration, as well as EC and pericyte hyperplasia in disease-affected SAT (Michelini et al., 2025). This supports the assumption that higher proliferation of these cell populations promotes hypervascularization marked by increased expression of platelet endothelial cell adhesion molecule 1 (*PECAM1*, also: *CD31*); (Al-Ghadban et al., 2019). This is also supported by the observation of higher numbers of *CD38*-and *ZNF423*-positive ASPC subsets that were described as committed intermediate preadipocytes (CPAs). *ZNF423* is an upstream regulator of *PPARG*, controlling the adipogenic commitment of early stromal progenitor cells (Table 2; Figure 3). They localize to the adipose tissue vasculature as a progenitor for both ECs and PCs. It could be postulated that these cells might play a role in the formation of hyperplastic lipedema affected adipose tissue through an endothelial-mesenchymal conversion (Gupta et al., 2012; Michelini et al., 2025), forming higher numbers of CD146 protein expressing progenitors in SAT of patients with lipedema, with lower adipogenic potential when differentiated *in vitro,* compared to cells from age matched individuals (Priglinger et al., 2017). The disease related formation of these cells could further be boosted by higher numbers of macrophages that secrete cytokines like TNFα, promoting gene expression of *CD146*. Although these cells are committed to the adipocyte lineage it was speculated that they might fail to fully differentiate to mature adipocytes, but the higher recruitment might lead to the expansion of fat mass in total (Priglinger et al., 2017; Strohmeier et al., 2022). In accordance to this it was speculated that the disease related changes in the microenvironment and the secretome of the lipedema-affected SVF directly increases leakiness of ECs causing endothelial barrier dysfunction, leading again to higher recruitment of immune cells already in early-stage lipedema (Strohmeier et al., 2022).

In summary, endothelial degeneration and vessel leakage could be core aspects of lipedema’s pathology, which, as a result, could contribute to increased recruitment of immune cells and changes in adipocyte development. An increased protein expression of pericyte marker CD146 and higher proportions of committed adipogenic progenitors in the endothelial microenvironment support the assumption that alterations in the vascular composition may contribute to cellular changes in the adipose tissue regions affected by lipedema (Figure 3). To further delineate lipedema-related effects on the development of APSP developmental trajectories, snRNA-seq or scRNA-seq analyses are warranted to analyze subset proportions as well as molecular and functional alterations in greater detail.

## Macrophages and immune cells in lipedema: links between inflammation and adipose tissue fibrosis

It is well established that lipedema is linked to inflammation and increased fibrosis in SAT-regions affected by lipedema. However, beyond macrophage phenotypical analyses, little is known about immune cell changes in adipose tissue of patients with lipedema. Activation of mast cells, which secrete high levels of histamine, has been reported (Bonetti et al., 2023). While the overall number of protein tyrosine phosphatase receptor type C (*PTPRC;* also: *CD45*)-expressing cells, including T-cells, B-cells, dendritic cells, eosinophils and natural killer cells was not affected by lipedema (Cifarelli et al., 2025), several studies describe an increased formation of crown-like structures around adipocytes in lipedema-affected SAT (Suga et al., 2009; Al-Ghadban et al., 2019). It was reported that mainly anti-inflammatory, M2-like macrophages are recruited to lipedema adipose tissue (Wolf et al., 2022; Kruppa et al., 2023; Vasella et al., 2023). While M2-like macrophages were the main type found in early, stage I lipedema, marked by high expression of scavenger receptor cysteine-rich type 1 protein M130 (*CD163*) and mannose receptor c-type 1 (*MRC1*, also: *CD206*), in later and more severe disease states, that are often accompanied by increasing BMI, a higher recruitment of pro-inflammatory macrophages was observed (Kruppa et al., 2023). This switch in macrophage phenotype might be due to dying adipocytes that occur in higher frequencies in obesity-linked chronic inflammation, and which is characterized by higher secretion of pro-inflammatory cytokines like TNFα that in turn could trigger additional tissue damage (Hotamisligil et al., 1993; Weisberg et al., 2003; Xu et al., 2003; Lumeng et al., 2007). Although it was reported that patients with lipedema suffer from systemic inflammation (Nankam et al., 2022), the higher recruitment of M2-like macrophages could also improve adipose tissue glucose uptake and insulin sensitivity, leading to a healthier blood lipid profile compared to BMI-matched, overweight/obese individuals (Torre et al., 2018; Nankam et al., 2022; Kruppa et al., 2023). Of note, the disease-related M2-like macrophage bias was observed exclusively in the lipedema-affected adipose tissue of the thigh region, but not in abdominal SAT that is thought to be unaffected by the disease. It remains to be determined if macrophage polarization, that clearly distinguishes early-stage lipedema from obesity, might serve as an early biomarker to improve lipedema diagnosis.

It should be noted that the descriptions of M1-and M2-like macrophages refer only to the endpoints of macrophage differentiation, and that numerous intermediate stages exist, covering the spectrum from pro- towards anti-inflammatory phenotypes, but until now no literature is available analyzing further macrophage subtypes in lipedema affected tissue. Among these, lipid-associated macrophages (LAMs), a subset of adipose tissue-resident macrophages were recently described, that might play a key role in adipose tissue remodeling and onset of fibrosis (Keophiphath et al., 2009; Chini et al., 2026). Concomitant with changes of adipocyte plasticity in relation to lipedema, this might also lead to pathological remodeling of the extracellular matrix (ECM), that was observed in lipedema in a stage-dependent manner, starting in early states of disease progression, before even adipocyte hypertrophy became evident (Kruppa et al., 2023). This is also evident from interstitial fibrosis and increased accumulation of laminin and type-VI collagen in adipose tissue samples of patients with lipedema (Al-Ghadban et al., 2020b). Whether quantitative analyses of such proteins could be integrated into everyday clinical practice as potential diagnostic biomarkers remains to be elucidated.

## Limitations and future perspectives

Research addressing lipedema, an adipose tissue-linked disease, has gained significant interest in recent years. It is by now well-established that lipedema is a disease that is clearly distinct from obesity, featuring lipedema-specific changes in the structure and function of SAT in disease-affected areas and might be reflected by a unique marker expression pattern. However, the number of available studies remains limited by varying study outcomes. Patient cohorts are typically comparably small and inclusion criteria and diagnostic procedures may vary considerably. Due to the variable age of onset and the progressive nature of lipedema, there are also significant differences in age and weight among patients at various stages of the condition (Kruppa et al., 2023; Straub et al., 2025). While comparator groups are frequently BMI- and age-matched, such parameters are not fully sufficient to investigate changes caused by lipedema in detail, for instance due to the altered adipose tissue distribution pattern that renders BMI comparisons between groups difficult. Medical histories could be expanded to include measures such as waist-to-height and waist-to-hip ratios, and the patients’ hormonal status should be assessed, as it was shown that these parameters are also influencing lipedema progression (Straub et al., 2025). A first step was taken within the research community through a Delphi Consensus methodology-based position paper (Kruppa et al., 2026), which could help to develop a standardized definition of lipedema symptoms and treatments. Further, in relation to the technical variabilities surrounding tissue collection and processing for cell cultures and molecular studies discussed in this review, it is recommended to use fresh frozen or freshly isolated cells when analyzing cellular development, dynamics, identities and composition, as cell culture in general affects cellular functions, which leads to significant differences in the interpretation of the results and thus creates the risk to draw contradictory and potentially misleading conclusions.

The adipose tissue cellular composition is highly variable and depends on both extracellular stimuli and cell-cell interactions. Gene expression and cytometric analyses only provide overviews about tissue dynamics in general, but do not consider heterogeneous distribution of cells throughout the adipose depots and the structural differences between different SAT layers. The publication of adipose tissue atlases that are based on single cell and single nucleus analyses can serve as platforms to analyze adipose tissue dynamics in health and under various pathological conditions (Zhong et al., 2025). As a next step, spatial transcriptomics methods could be integrated with single cell/nucleus-based technologies to better understand the relationship between tissue organization and structure (Backdahl et al., 2021; Massier et al., 2023). This could serve as a basis for understanding lipedema-induced changes in cellular networks and the local niches and microenvironments and could help determine whether patients with lipedema experience cellular reprogramming or cellular rearrangements. However, even when using high-resolution analytical technologies, caution must be exercised when interpreting the results and comparing different studies, as varying results can arise from technical variations in bioinformatic analysis pipelines, e.g., in filtering steps, resolution, cluster definitions, and batch correction steps.

Several studies now agree that lipedema-affected SAT shows a disease specific immune reaction that is distinct from macrophage patterns in adipose tissue of healthy individuals or patients with obesity, where mainly anti-inflammatory type 2 like macrophages are recruited to the tissue surrounding necrotic adipocytes and the endothelial niche (Al-Ghadban et al., 2019; Felmerer et al., 2020b; Wolf et al., 2022; Kruppa et al., 2023). Apart from this, little is known about macrophage subtype composition, other immune cells like T- and B-cells and their compositional changes in adipose tissue affected by lipedema. Although it was reported that the total amount of T- and B cells is not affected by the disease, lipedema may still impact on their respective functions and subtype distributions. As no study currently reports altered composition of macrophage- or T-cell subtypes in lipedema at single-cell resolution or thought flow cytometric analyses, this aspect of immune cell biology remains an unexplored area requiring further investigation.

An altered immune response with an imbalanced macrophage ratio might also trigger vascular dysfunction due to an altered cytokine profiles in disease-affected tissue regions. The vascular dysfunction might lead to an amplified immune response, resulting in sustained chronic inflammation, dysfunctional angiogenesis, and increased vascular permeability (Al-Ghadban et al., 2019; Allen et al., 2020; Felmerer et al., 2020b; Michelini et al., 2025). In agreement with this, interstitial fluid is known to accumulate in SAT of patients with lipedema, leading to a hypoxic microenvironment with altered adipocyte and ASPC functions that could trigger extracellular matrix remodeling and pathological cytokine signaling (Allen et al., 2020; Crescenzi et al., 2023; Ernst et al., 2023). To clearly define lipedema-related phenotypes and distinguish them from phenotypes related to adipose tissue-linked pathologies and comorbidities, it will be necessary to combine *in vivo* imaging techniques with high-resolution structural and cellular biological analyses, considering cohorts with precisely matched comparator groups, while taking several anthropometric factors into account.

## Conclusion

Although research on lipedema has received substantial attention in recent years, many aspects of the disease remain unclear or controversial. Patient inclusion and exclusion criteria, matching comparator cohorts, as well as sample processing and experimental design remain critical factors that make it challenging to compare different studies. Improved characterization and diagnosis of lipedema through high-sensitivity single cell analyses could help further this process, but also carries a risk for misinterpretation, as some potential diagnostic markers can be attributed to different cell populations. For example, preliminary data indicate that there is a functional reprogramming of mature adipocytes, but the data could also indicate changes in disease-affected adipocyte development, where the functionalities of adipose tissue-resident progenitors is altered. The impact of lipedema on the complex interactions among the various cell populations in the SAT, as well as its systemic effects, is still incompletely understood. Further studies will be needed to potentially identify one or more biomarkers for the disease that could not only facilitate diagnosis but also help to better understand disease stage and progression and develop effective treatments beyond liposuction.
